# Association between institutional procedural preference and in-hospital outcomes in laparoscopic surgeries; Insights from a retrospective cohort analysis of a nationwide surgical database in Japan

**DOI:** 10.1371/journal.pone.0193186

**Published:** 2018-03-05

**Authors:** Hiroaki Miyata, Masaki Mori, Norihiro Kokudo, Mitsukazu Gotoh, Hiroyuki Konno, Go Wakabayashi, Hisahiro Matsubara, Toshiaki Watanabe, Minoru Ono, Hideki Hashimoto, Hiroyuki Yamamoto, Hiraku Kumamaru, Shun Kohsaka, Tadashi Iwanaka

**Affiliations:** 1 National Clinical Database, Tokyo, Japan; 2 Department of Health Policy and Management, Keio University, Tokyo, Japan; 3 The Japanese Society of Gastroenterological Surgery, Tokyo, Japan; 4 Department of Gastroenterological Surgery, Graduate School of Medicine, Osaka University, Osaka, Japan; 5 Japan Surgical Society, Tokyo, Japan; 6 National Center for Global Health and Medicine, Tokyo, Japan; 7 Osaka General Medical Center, Osaka, Japan; 8 Hamamatsu University School of Medicine, Hamamatsu, Japan; 9 Department of Surgery, Ageo Central General Hospital, Saitama, Japan; 10 Department of Frontier Surgery, Graduate School of Medicine, Chiba University, Chiba, Japan; 11 Department of Surgical Oncology, Graduate School of Medicine, The University of Tokyo, Tokyo, Japan; 12 Department of Cardiac Surgery, The University of Tokyo, Tokyo, Japan; 13 Department of Health and Social Behavior, School of Public Health, The University of Tokyo, Tokyo, Japan; 14 Department of Healthcare Quality Assessment, Graduate School of Medicine, The University of Tokyo, Tokyo, Japan; 15 Department of Cardiology, Keio University School of Medicine, Tokyo, Japan; 16 Bureau of Saitama Prefectural Hospitals, Saitama, Japan; CHU Clermont-Ferrand, FRANCE

## Abstract

**Objective:**

To assess the use of laparoscopic surgeries (LS) and the association between its performance and hospitals’ preference for LS over open surgeries.

**Summary background data:**

LS is increasingly used in many abdominal surgeries, albeit both with and without solid guideline recommendations. To date, the hospitals’ preference (LS vs. open surgeries) and its association with in-hospital outcomes has not been evaluated.

**Methods:**

We enrolled patients undergoing 8 types of gastrointestinal surgeries in 2011–2013 in the Japanese National Clinical Database. We assessed the use of LS and the occurrences of surgery-related morbidity and mortality during the study period. Further, for 4 typical LS procedures, we assessed the hospitals’ preference for LS by modeling the propensity to perform LS (over open surgeries) from patient-level factors, and estimating each institution’s observed/expected (O/E) ratio for LS use. Institutions with O/E>2 were defined as LS-dominant. Using hierarchical logistic regression models, we assessed the association between LS preference and in-hospital outcomes.

**Results:**

Among 1,377,118 patients undergoing gastrointestinal procedures in 2,336 participating hospitals, use of LS increased in all 8 procedures (35.1% to 44.7% for distal gastrectomy (DG), and 27.5% to 43.2% for right hemi colectomy (RHC)). Those operated at LS-dominant hospitals were at an increased risk of operative death (OR 1.83 [95%CI, 1.37–2.45] for DG, 1.79 [95%CI, 1.43–2.25] for RHC) compared to standard O/E level hospitals (0.5≤O/E<2.0).

**Conclusions:**

LS use widely increased during 2011–2013 in Japan. Facilities with higher than expected LS use had higher mortality compared to other hospitals, suggesting a need for careful patient selection and dissemination of the procedure.

## Background

Since its introduction in the late 1980s, laparoscopic surgery (LS) has been implemented in various types of abdominal surgeries. In Japan, more than 478,000 abdominal surgeries were performed in 2012, of which 30.1% were LS. The rate of LS had increased by 4.5% from the previous year. With the worldwide trend of patients’ preferences for less invasive procedures, LS will gain even more popularity in the near future. Consequently, there has been a high demand for investigations on the overall clinical impact of its rapid growing use in order to ensure patients’ safety.

Currently, LS are gaining the biggest popularity in the area of surgeries that have established evidence and solid guideline recommendations (e.g., laparoscopic distal gastrectomy and colectomy) [1–4]. However, the LS operations also seem to have expanded to areas less clinically established (e.g., Total gastrectomy) [5], where data from clinical trials are sparse and the clinical indication is ambiguous. Therefore, on-going data registrations along with systematic feedback to the operators is essential. Furthermore, the safety of LS has attracted general public’s attention in Japan due to a news coverage that reported high incidence of adverse events for LS at a particular institution.

In this study, we assessed the data from a nation-wide clinical registry to provide materials for further discussion. The National Clinical Database (NCD) is Japan’s nationwide web-based clinical data entry system linked to surgical board certifications. As an academic consortium of professional surgical societies, we performed analyses on the overall clinical impact of rapid implementation of LS. Specifically, we set a protocol to describe: (1) the absolute number and ratio of LS vs. all abdominal surgeries for typical surgical procedures; (2) hospital-level association between procedural volume and clinical outcome of LS; and (3) the differences in the patient outcomes based on the institutional preference to LS over open abdominal surgery.

## Methods

### Data source

The data registration began in January, 2011. Over 4,200 institutions report to the NCD, and approximately 1,400,000 cases are registered annually, providing data on more than 95% of surgical procedures across the nation. Variable definitions of NCD are equivalent to those used by surgical societies that maintain advanced clinical registry, such as The Society of Thoracic Surgeons (STS) and the American College of Surgeons’ National Surgical Quality Improvement Program (ACS NSQIP), and it allows detailed and internationally comparable analyses [6–13]. NCD also puts high importance on data completeness and the quality of data like those two forerunners: Validation methods such as auditing, site-visiting, and education are routinely performed.

We conducted this retrospective cohort study using a clinical registry for gastroenterological surgery in Japan which was established as a division of NCD in 2011 in collaboration with the Japanese Society of Surgery (JSS), Japanese Society of Gastroenterological Surgery (JSGS), Japanese society of Hepato-Biliary-Pancreatic Surgery, Japan Esophageal Society, Japanese Gastric Cancer Association, Japanese Society for Cancer of the Colon and Rectum, Liver Cancer Society Group of Japan, Japan Pancreas Society, Japan Society for Endoscopic Surgery, and Japanese Society for Abdominal Emergency Medicine. [14] All member institutions of the JSS and JSGS are required to submit surgical cases to NCD for board certification purposes. [15]

### Data completeness

NCD ensures data traceability by tracking the staff who approve the data along with data-entry personnel at the participating institutions. It also validates the data’s consistency via random inspections of participating institutions. All variables and definitions for NCD data are systematically defined and accessible to the participants on database website (http://www.ncd.or.jp/); the database administrators also provide e-learning systems to teach the participants how to input consistent data. In addition, the administrative office members answer all inquiries regarding data entry. Frequently Asked Questions can be accessed at the organization website.

Data quality standards need to be met before a local dataset become part of the aggregated national analytic dataset. Data were cleaned and maintained independently by the Department of Healthcare Quality Assessment, The University of Tokyo, Tokyo, Japan, which produces annual site-specific reports to JSGS participant hospitals for outcome analyses and quality improvement. The registry employs an electric Web-based data collection system that does not permit missingness for the majority of the data components at registration. Therefore, prevalence of missing data is extremely low; a small number of records with missing age (or out of range), sex, or 30-day mortality were considered to have arisen from system error, and were excluded from the study population.

### Ethical consideration

Japan Surgical Society established an ethics committee that includes members of the Japanese Surgical Society ethics board, lawyers, patient representatives and experts on information security. The committee considered the ethical propriety of the entire initiative, approved it and made the review process public on the Japan Surgical Society website. [16] The use of the data from the registry for retrospective observational studies were approved by the committee, and individual written or verbal informed consent was waived because of the retrospective design.

### Inclusion/Exclusion criteria

We enrolled in the study cohort 1,377,118 patients undergoing abdominal surgeries between January 1, 2011 and December 31, 2013 at 2,336 hospitals throughout Japan, registered in the NCD. Patients who declined to have their records entered in the NCD and a small number of records with missing data on core data components including patients’ age, sex, or 30-day survival were excluded.

Detailed clinical variables such as the laboratory data (e.g. total bilirubin, aspartate amino transferase, alanine aminotransferase) and operative morbidities (e.g. wound events, respiratory events, urinary tract events, central nervous system, systemic sepsis) were requested to be entered for eight procedures: esophagostomy; total gastrectomy/distal gastrectomy (TG/DG); right hemicolectomy (RHC); low anterior resection (LAR); hepatectomy performed on >1 segment, except the lateral segment; pancreaticoduodenectomy; and operation for acute generalized peritonitis. For the present study, the demographic and outcome information along with the volume-outcome relationship for TG/DG [7,13], RHC [9], and LAR [10] were analyzed since these four types of surgeries were frequently performed via both open and laparoscopic approaches.

### Outcome measures

The outcome measures of this study were 30-day mortality as well as operative mortality. The former was defined as death within 30 days after surgery regardless of the patient’s hospitalization status. The latter was defined as death within 30 days or during the index hospitalization regardless of the length of the hospital stay (up to 90 days).

### Statistical analysis

We first assessed the time trend in the proportion of LS surgeries among all surgeries by type of surgical procedure, by estimating the proportion in each quarter starting from the 1^st^ quarter of 2011 up to the 4^th^ quarter of 2013. We evaluated hospital procedural volume for each hospital averaging over a 3-year period (2011–2013). Secondly, we described number of surgery, observed mortality and complication rate in both total surgeries and LS, biannually. Expected mortality and complication rate, and their observed/expected (O/E) ratio were calculated using previously established risk models for each surgical procedure (Table 1) [7, 9, 10, 13]. The discriminatory performance of these risk models in the current population measured by c-statistics were 0.84, 0.83, 0.89 and 0.83 for DG/TG, RHC and LAR, respectively.

**Table 1:** Risk models for each surgical procedure.

	Variables	Reference
**Distal gastrectomy** **(C-statistics 0.831)**	Age, ADL, Respiratory distress, Previous cerebrovascular disease, Weight loss, Ascites, Disseminated cancer, Chronic steroid use, Emergent surgery, ASA, White blood cells, Hematocrit, Platelets, Serum albumin, Aspartate aminotransferase, Alkaline phosphatase, Total bilirubin, Creatinine, Serum Na, PT-INR, APTT	[13]
**Total gastrectomy** **(C-statistics 0.824)**	ASA score, Disseminated cancer, Alkaline phosphatase, Total bilirubin, Preoperative dialysis, Pancreaticosplenectomy, White blood cell count, ADL, PT-INR, Cerebrovascular accident, Ascites, Respiratory distress, Aspartate aminotransferase, Status Emergent, White blood cell count, Weight loss, Sodium, Albumin, Hematocrit, Age	[7]
**Right hemicolectomy** **(C-statistics 0.891)**	Previous PVD surgery, Cancer with multiple metastases, ASA, AST, Platelet, Preoperative dialysis, ADL, Blood urea nitrogen, Congestive heart failure, Chronic steroid use, Emergent surgery, Sodium, Sepsis, Weight loss, Cancer metastasis relapse, White blood cell, Total bilirubin, Ascites, Albumin, Hematocrit, PT-INR, Age	[9]
**Low anterior resection** **(C-statistics 0.77)**	Age, Sex, Respiratory distress, ADL, Ascites, Previous surgery for PVD, Disseminated cancer, Preoperative transfusions, BMI, Serum creatinine, Hemoglobin, Hematocrit, Platelet, Serum albumin, AST, Na	[10]

Additionally, we analyzed the relationship between annual procedure volume and surgical mortality. In the volume-outcome analysis, the volume was modeled as a continuous variable. For display purposes, the O/E mortality ratio was categorized based on the annual hospital procedural volume. A hierarchical mixed-effects logistic regression model was used to test the effect of hospital procedural volume on operative mortality accounting for clustering of patients at the hospital level. These analyses included in the model previously identified clinical risk factors [7,9,10,13], calendar period, indicator for the procedure being laparoscopic or open surgery, and hospital procedural volume as fixed effects, and random intercepts for the sites. We also examined the effect of hospital LS volume on the LS operative mortality using a similar method.

In order to assess the hospitals’ preference on performing LS over open surgery for each procedure, we developed 4 multivariable logistic regression models predicting LS procedure over open surgery from above mentioned outcome predictors using forward step-wise selection with a *p* value of 0.05 for inclusion and 0.10 for exit. These regression analyses included the following patient level variables in the model: age, sex, use of emergency ambulance, urgency of the emergent surgery, diabetes, smoking, alcohol consumption, respiratory distress, activities of daily living, use of preoperative mechanical ventilation, comorbidities (chronic obstructive pulmonary disease, preoperative pneumonia, ascites, esophageal varices, hypertension, congestive heart failure, myocardial infarction, angina, previous percutaneous coronary intervention, previous cardiac surgery, previous peripheral vascular disease surgery, peripheral vascular disease, acute renal failure, preoperative dialysis, cerebrovascular disease, cerebrovascular accident, disseminated cancer, open wound, chronic steroid use, weight loss, bleeding disorder, systemic sepsis), preoperative transfusion, preoperative chemotherapy, preoperative radiation therapy, American Society of Anaesthesiologists physical status classification, non-tumor bearing status, brinkman index, body mass index, and laboratory data (white blood cell, hemoglobin, hematocrit, platelet, albumin, blood urea nitrogen, creatinine, total bilirubin, aspartate transaminase, alkaline phosphatase, serum sodium, C-reactive protein, prothrombin time, activated partial thromboplastin time).

Using these models developed from all patients receiving the procedure in the cohort, we estimated the number of patients expected to undergo LS surgery at each hospital based on patient case mix; i.e. by summing up the predicted probability of LS for the patients at the hospital. The selected variables and c-statistics in the model for each procedure were described in Table 2. We defined hospitals’ preference score for LS as the ratio between the observed and the expected number of LS procedure; preference score in O/E ratio >1.0 indicated that they more readily adopted LS than expected, while ratios <1.0 indicated that the hospitals adopted LS less frequently than expected. We categorized this hospital preference for LS into three groups based on O/E ratio: O/E ≥2 (active group), 0.5≤O/E<2.0 (standard group), and O/E<0.5, and conducted a hierarchical mixed-effects logistic regression analysis to assess the association between the O/E ratio category and operative mortality, including in the model, expected mortality for each patient calculated by previous risk model, procedure period, hospital procedural volume, and preference score category as fixed effects, and random intercepts for the sites. We also did a similar hierarchical logistics regression analysis in which the preference score was treated as a continuous variable. In the model, the preference category was now replaced by the actual O/E score, while O/E score <1 was further replaced by 1, therefore treating the cases performed at these hospitals as the reference. To assess the association between the LS preference and outcome separately for LS or OS procedures as well as by hospital case volume (<20 vs. 20 and over), we conducted additional analyses in the corresponding subgroups. All statistical analyses were performed using SAS, version 9.2 (SAS Institute, Cary, NC, USA) and SPSS, version 20.0 (SPSS, Chicago, IL, USA). *P* < 0.05 was considered statistically significant.

**Table 2:** Models for LS propensity in each surgical procedure.

	Variables
**Distal gastrectomy** **(C-statistics 0.676)**	Age, Sex, Ambulance transport, Emergent surgery, Smoking, Alcohol, Respiratory distress, ADL, COPD, Ascites, Hypertension, Congestive heart failure, PVD, Previous cerebrovascular disease, Disseminated cancer, Chronic steroid use, Weight loss, Preoperative transfusions, Preoperative chemotherapy, Sepsis, ASA, Brinkman index, BMI, Creatinine, Hemoglobin, Hematocrit, Platelets, Serum albumin, Alkaline phosphatase, Blood urea nitrogen, Serum Na, CRP, PT-INR, White blood cells, APTT
**Total gastrectomy** **(C-statistics 0.669)**	Age, Sex, Ambulance transport, Emergent surgery, Alcohol, COPD, Ascites, Hypertension, Disseminated cancer, Weight loss, Preoperative transfusions, Preoperative chemotherapy, ASA, No tumor, Non-cancer surgery, Hemoglobin, Hematocrit, Platelets, Albumin, Total bilirubin, Alkaline phosphatase, Blood urea nitrogen, Serum Na, CRP, PT-INR, White blood cell count
**Right hemicolectomy** **(C-statistics 0.706)**	Age, Sex, Ambulance transport, Emergent surgery, Smoking, Alcohol, Respiratory distress, ADL, Preoperative ventilation, Ascites, Hypertension, Congestive heart failure, Disseminated cancer, Open wound, Weight loss, Bleeding disorder, Preoperative transfusions, Preoperative chemotherapy, Sepsis, No tumor, Non-cancer surgery, Brinkman index, BMI, Hemoglobin, Platelets, Albumin, Alanine aminotransferase, Alkaline phosphatase, Blood urea nitrogen, Serum Na, CRP, PT-INR, White blood cells, APTT
**Low anterior resection** **(C-statistics 0.626)**	Age, Ambulance transport, Emergent surgery, Smoking, Alcohol, ADL, Ascites, Hypertension, Congestive heart failure, Previous cardiac surgery, Disseminated cancer, Weight loss, Preoperative transfusions, Preoperative chemotherapy, Preoperative radiotherapy, Non-cancer surgery, Brinkman index, Hemoglobin, Platelet, Albumin, Total bilirubin, Alkaline phosphatase, Blood urea nitrogen, Serum Na, CRP, PT, PT-INR, White blood cells, APTT

## Results

### LS expansion and performance

**Fig 1:**
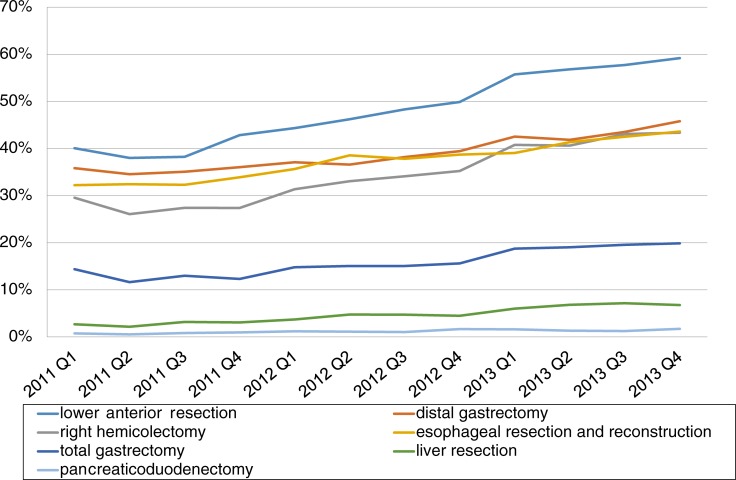
Proportion of LS among all surgeries by surgical procedure type over time. Q, quarter.

Fig 1 presents the time trend of the LS implementation from the first quarter of 2011 to the fourth quarter of 2013 for the registered abdominal surgeries. During the 3-year period, the use of LS had increased substantially; the proportion of LS surgery increased by 19.1 percentage points for LAR, by 13.8 percentage points for RHC, and by 10.8 percentage points for esophageal resections and reconstructions.

Tables 3 and 4 describe the observed operative mortality, expected operative mortality based on our prediction model, and the O/E ratio for the four types of surgical procedures by year periods (first half of year 2011 until latter half of 2013), among both open surgery and LS patients, and among LS patients alone. The operative mortality risk, the risk of complications, the observed/expected mortality ratio, and the observed/expected complication ratio were consistent over time for all four LS. The total surgical operative mortality ratio and observed/expected mortality ratio were also consistent.

**Table 3:** Descriptive statistics over time for total surgical procedure.

Procedure	Time period	Total (Open surgery and LS)			
		Num. of surgery	Observed mortality	Expected mortality	O/E ratio (mortality)
Distal gastrectomy	1^st^ half 2011	13,966	1.20%	1.16%	1.03
	2^nd^ half 2011	17,169	1.07%	1.15%	0.93
	1^st^ half 2012	16,889	1.04%	1.08%	0.96
	2^nd^ half 2012	17,882	1.00%	1.06%	0.94
	1^st^ half 2013	17,391	1.07%	1.10%	0.97
	2^nd^ half 2013	18,184	1.09%	1.09%	1.00
Total gastrectomy	1^st^ half 2011	8360	2.25%	2.21%	1.02
	2^nd^ half 2011	10,062	2.22%	2.37%	0.94
	1^st^ half 2012	10,100	2.34%	2.25%	1.04
	2^nd^ half 2012	10,731	2.32%	2.06%	1.13
	1^st^ half 2013	9130	2.27%	2.14%	1.06
	2^nd^ half 2013	9614	2.23%	2.16%	1.03
Right hemicolectomy	1^st^ half 2011	7697	2.27%	2.22%	1.02
	2^nd^ half 2011	9775	2.10%	2.12%	0.99
	1^st^ half 2012	9672	2.13%	1.92%	1.11
	2^nd^ half 2012	10,886	2.14%	1.73%	1.24
	1^st^ half 2013	10,340	2.50%	1.68%	1.49
	2^nd^ half 2013	10,876	2.11%	1.59%	1.32
Low anterior resection	1^st^ half 2011	6992	0.94%	0.84%	1.12
	2^nd^ half 2011	8265	0.71%	0.84%	0.85
	1^st^ half 2012	9009	0.64%	0.79%	0.81
	2^nd^ half 2012	9145	0.70%	0.73%	0.96
	1^st^ half 2013	9063	0.63%	0.70%	0.90
	2^nd^ half 2013	9158	0.87%	0.70%	1.25

LS, laparoscopic surgeries; O/E, observed/expected

**Table 4:** Descriptive statistics regarding time trend for LS.

Procedure	Time period	LS								
		Number of LS	LS proportion	30day mortality	Observed mortality	Expected mortality	O/E ratio (mortality)	Observed complication	Expected complication	O/E ratio (complication)
Distal gastrectomy	1^st^ half 2011	4901	35.1%	0.20%	0.43%	0.63%	0.68	10.59%	12.82%	0.83
	2^nd^ half 2011	6105	35.6%	0.20%	0.46%	0.61%	0.75	11.99%	12.83%	0.93
	1^st^ half 2012	6222	36.8%	0.16%	0.43%	0.59%	0.74	11.35%	12.67%	0.90
	2^nd^ half 2012	6941	38.8%	0.19%	0.30%	0.59%	0.51	12.36%	12.83%	0.96
	1^st^ half 2013	7337	42.2%	0.23%	0.42%	0.62%	0.68	11.12%	12.95%	0.86
	2^nd^ half 2013	8120	44.7%	0.31%	0.53%	0.63%	0.84	11.86%	12.96%	0.92
Total gastrectomy	1^st^ half 2011	1071	12.8%	0.28%	0.37%	1.12%	0.33	17.74%	19.53%	0.91
	2^nd^ half 2011	1273	12.7%	0.47%	1.10%	1.38%	0.80	20.97%	20.27%	1.03
	1^st^ half 2012	1508	14.9%	0.27%	0.66%	1.26%	0.53	16.51%	19.03%	0.87
	2^nd^ half 2012	1646	15.3%	0.55%	0.97%	1.14%	0.85	20.41%	19.54%	1.04
	1^st^ half 2013	1725	18.9%	0.75%	1.39%	1.21%	1.15	18.55%	19.43%	0.95
	2^nd^ half 2013	1895	19.7%	0.42%	0.69%	1.17%	0.59	18.68%	19.63%	0.95
Right hemicolectomy	1^st^ half 2011	2113	27.5%	0.28%	0.43%	0.86%	0.50	11.22%	14.18%	0.79
	2^nd^ half 2011	2678	27.4%	0.26%	0.60%	0.91%	0.66	12.21%	14.14%	0.86
	1^st^ half 2012	3117	32.2%	0.29%	0.55%	0.79%	0.69	11.20%	13.49%	0.83
	2^nd^ half 2012	3774	34.7%	0.26%	0.48%	0.77%	0.62	13.22%	13.45%	0.98
	1^st^ half 2013	4208	40.7%	0.33%	0.69%	0.75%	0.92	12.90%	13.71%	0.94
	2^nd^ half 2013	4701	43.2%	0.32%	0.53%	0.71%	0.75	10.21%	13.46%	0.76
Low anterior resection	1^st^ half 2011	2719	38.9%	0.26%	0.59%	0.60%	0.98	19.46%	20.72%	0.94
	2^nd^ half 2011	3348	40.5%	0.33%	0.63%	0.63%	1.00	20.97%	21.03%	1.00
	1^st^ half 2012	4079	45.3%	0.25%	0.44%	0.60%	0.74	18.68%	20.01%	0.93
	2^nd^ half 2012	4490	49.1%	0.38%	0.49%	0.55%	0.89	20.11%	20.12%	1.00
	1^st^ half 2013	5101	56.3%	0.27%	0.47%	0.57%	0.83	18.09%	20.10%	0.90
	2^nd^ half 2013	5355	58.5%	0.26%	0.73%	0.62%	1.17	17.54%	20.25%	0.87

LS, laparoscopic surgeries; O/E, observed/expected.

#### Volume-outcome of LS vs open procedures

**Fig 2:**
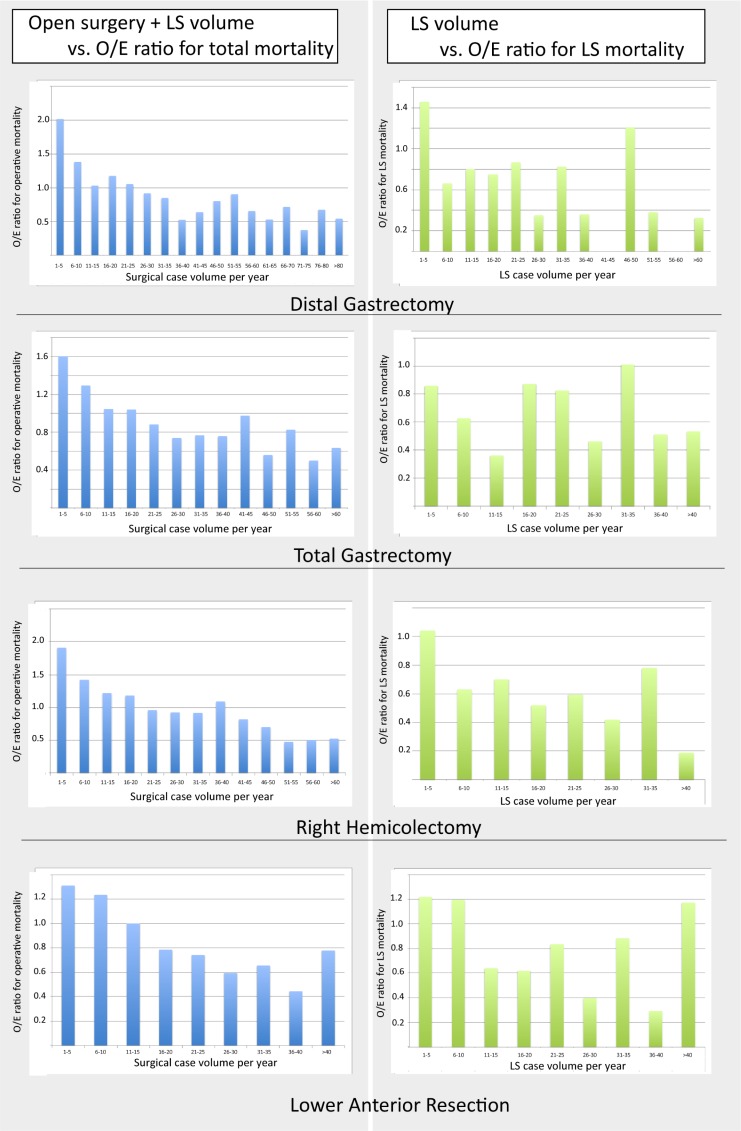
Relationship between annual procedure volume vs. observed/expected operative mortality ratio for all surgeries and for LS by procedure. O/E, observed/expected.

**Fig 3:**
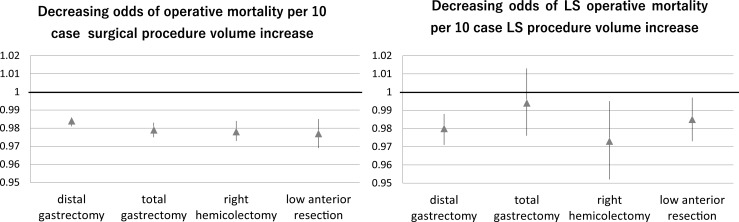
Association between a 10 case increase in surgical and LS volume and odds of operative mortality. The point estimates of the odds ratio (closed triangle) and the 95% confidence interval are demonstrated.

The annual hospital surgical volume for each of the four procedures, along with the O/E mortality ratio by the total surgical volume, and the O/E LS operative mortality ratio per the hospital’s LS volume category are described in Fig 2. The adjusted operative mortality declined significantly as a function of the hospital procedural volume (Fig 3) after adjusting for the preoperative clinical risks, the calendar period of surgery, the treatment indication, and the patient clustering within the centers. Although the relationship between the hospital procedural volume and the risk-adjusted mortality was present for all four types of LS, there were wide variances in the results. The distribution of the hospital annual volume for each surgery is demonstrated in S1 Fig; notably, a large majority of hospitals are categorized into the low volume groups.

### Institutional preference for LS and patient outcomes

**Fig 4:**
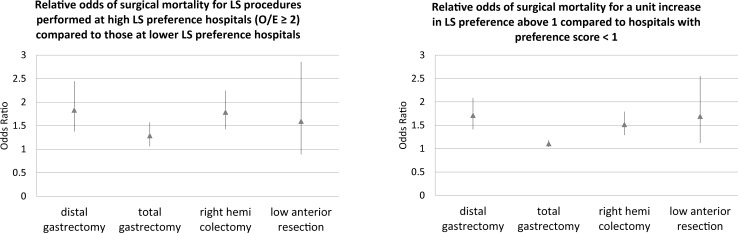
Increased odds of operative mortality associated with increased LS institutional preference score. The point estimates of the odds ratio (closed triangle) and the 95% confidence interval are demonstrated.

We describe the distribution of the hospital level LS implementation rate (proportion of each procedure that is performed laparoscopically) for each of the four procedures in S2 Fig, among the hospitals with annual procedure volume ≥10. We also present the distribution of the observed/expected ratio for number of LS surgeries which represents the hospitals’ preference for LS in S3 Fig. The descriptive statistics for the hospital group by the hospital LS preference category are displayed in Table 5. LS dominant hospitals were more likely to be small volume hospitals compared to standard O/E level hospitals (0.5≤O/E<2.0). The number of those treated at high LS preference hospitals were 5507 for DG, 9324 for TG, 5376 for RHC, and 1160 for LAR. In Fig 4, we show the results from the hierarchical logistic regression analysis evaluating the relative odds of operative mortality for being treated at higher LS preference score hospitals. Even after making adjustments, the risk of operative mortality was significantly higher for active implementation hospitals in the three procedures (OR 1.83 [95%CI, 1.37–2.45] for DG, 1.29 [95%CI, 1.06–1.57] for TG, 1.79 [95%CI, 1.43–2.25] for RHC, 1.60 [95%CI 0.89–2.86] for LAR). Also, when treating the preference score as continuous when above 1, a unit increase in the preference score was associated with significantly higher odds of operative death in all four procedures after adjusting for the preoperative clinical risks, period of surgery, treatment preference, hospital volume, and patient clustering within the centers. The results were not different between low volume hospitals (< 20 cases every year) and others (≥ 20 cases every year) (Table 6). High LS preference was associated with higher mortality among the cases undergoing LS procedures for distal gastrectomy and total gastrectomy; interestingly, the increase in the risk was higher among the OS patients for in right hemicolectomy.

**Table 5:** Descriptive statistics for each treatment indication hospital group.

surgery type	variables	low LS preference hospital (O/E under 0.5)	middle LS preference hospital (O/E 0.5–2)	high LS preference hospital (O/E 2 and over)
distal gastrectomy	number of hospital	976	982	119
	number of patient	24,248	71,726	5507
	annual volume	4 (1.3–10.3)	17.0 (7.7–32.4)	6 (2.3–18.7)
	length of stay	24.5 ± 18.4	21.7 ± 16.9	21.6 ± 20.0
	operative mortality	1.3%	0.8%	1.1%
total gastrectomy	number of hospital	1267	439	255
	number of patient	27,770	20903	9324
	annual volume	3.7 (1.3–9.3)	12.0 (6.0–22.7)	7 (2.7–17.3)
	length of stay	29.0 ± 21.4	26.7 ± 20.4	27.0 ± 21.1
	operative mortality	2.4%	2.0%	2.0%
right hemi colectomy	number of hospital	938	955	151
	number of patient	16,716	37,154	5376
	annual volume	3.3 (1.3–8.0)	9.7 (4.7–18.3)	9.3 (3.0–18.3)
	length of stay	25.7 ± 20.1	22.8 ± 18.5	23.1 ± 20.2
	operative mortality	2.3%	2.0%	2.3%
low anterior resection	number of hospital	833	1079	62
	number of patient	12,945	37,527	1160
	annual volume	2.7 (1.0–6.5)	7.6 (3.7–15.3)	3.0 (1.0–9.0)
	length of stay	27.2 ± 20.1	25.0 ± 19.8	26.6 ± 19.6
	operative mortality	0.8%	0.7%	1.3%

Annual volume and length of stay were expressed as the median (interquartile range) and mean ± standard, respectively. O/E, observed/expected.

**Table 6:** Subgroups analyses for relative odds of surgical mortality in LS dominant hospital (O/E ≥2) compared to standard LS preference hospitals (0.5≤O/E<2) by surgical type (LS vs. OS) and by hospital volume.

		Laparoscopic surgery			Open surgery		
		OR	95%CI		OR	95%CI	
Distal gastrectomy		2.08	1.45	2.98	1.44	0.86	2.40
Total gastrectomy		1.89	1.12	3.18	1.17	0.94	1.46
Right hemicolectomy		1.48	0.96	2.29	1.81	1.37	2.38
Low anterior resection		1.46	0.77	2.77	3.17	0.69	14.60
		Hospital volume ≥ 20 cases/year			Hospital volume < 20 cases/year		
		OR	95%CI		OR	95%CI	
Distal gastrectomy		1.68	1.10	2.56	1.65	1.10	2.47
Total gastrectomy		1.21	0.88	1.65	1.31	1.01	1.69
Right hemicolectomy		1.96	1.34	2.86	1.72	1.29	2.28
Low anterior resection		1.53	0.48	4.85	1.62	0.82	3.21

## Discussion

### Current status of LS in Japan

The main objective of the present study was to provide an overview of the LS implementation and performance in Japan. From 2011 to 2013, the use of LS had increased substantially. The mortality and standardized mortality rates of LS as well as the rates for all abdominal surgeries had been fairly constant over this time.

Our results provide evidence for making the decisions regarding appropriate therapeutic indications and detailed information on the impact of each surgical procedure. It is worth noting that similar decreasing trends in mortality by volume were observed in all types of LS, in both procedures with indications confirmed by clinical guidelines (DG/TG or RHC) and procedures with less established indications (LAR or surgeries of the pancreas and liver region). The primary concern is the effect of volume on the outcome for the use of the procedure in cases where therapeutic indications have not been clarified. At the same time, the current study’s findings suggested that the preoperative risk of mortality and the complications associated with LS are consistently low nationwide.

### Safe implementation of LS

The therapeutic indications for laparoscopic surgery have not changed substantially over the study period; however, there are large differences in its implementation across the facilities. The social benefits of a laparoscopic approach should be considered with the future expansion of the therapeutic indications of LS. Our risk-adjusted analysis showed that the annual number of cases of the overall surgical volume was consistently associated with the mortality risk from that procedure (i.e., the higher the volume, the lower the mortality). For the safe implementation of LS, a certain level of case experience is required [17–19]. In addition, our analysis indicated that there were variations in the impact of the annual number of LS cases on perioperative mortality across procedures. This may be a consequence of poor choice in procedure and inappropriate training environments during the introductory period in various types of LS [20,21].

Our findings also indicate that the careless expansion of LS volume is not desirable in order to perform the surgeries safely. Facilities with a few cases constitute a high risk with regard to the patients’ safety. According to our data, facilities that are excessively active in adopting LS are associated with higher risks, even after adjusting for the annual facility case volume and patient risk factors using appropriate statistical modeling; this suggests that overutilization of LS may be associated with increased risk. The impact of this increased risk is substantial, given the number of patients treated at these hospitals, as well as the baseline peri-operative mortality for these surgical procedures; our results suggest that if the preference at the high preference hospitals are reduced to the level of medium preference hospitals, through training and education for patient selection as well as appropriate between-hospital collaborations, we can expect a reduction of 1 operative mortality from the numbers of procedures as small as 153 for DG, 177 for TG, 66 for RHC, and 241 for LAR. Between facilities that are actively adopting LS and those that are not, cases undertaken at the former tended to have reduced bleeding; however, there was a trend toward a higher incidence of re-operation and re-hospitalization (data not shown). This finding is in agreement with the existing reports on LS. It would be desirable to learn techniques at a facility with a certain amount of total case experiences (i.e., both open and laparoscopic surgeries).

However, for LAR, in which a trend toward excessive adoption was not a significant risk, descriptive statistics showed differences; however, the standardized operation ratio (i.e. the LS preference score) did not show a large variation. This may be related to the fact that the number of facilities that actively implement the procedure was small. The increasing use of a technique during an introductory period is an important factor in terms of advancing medical technology; however, increased use presents some risks. Thus, it is necessary to make careful decisions to ensure that the surgery can be safely performed. To establish a system that can provide a higher quality of LS, it will be necessary to develop an educational system that assumes case experiences [19,20], including abdominal surgeries, and an implementation system that controls treatment indications with safety considerations.

### For future improvement of surgical safety

Treatment results may be unsatisfactory after accounting for pre-operative severity even at facilities with a high enough number of cases. In such facilities, a support is necessary to improve the treatment results. In Japan, cardiac surgery was the first field to initiate a benchmarking process. Instead of simply providing feedback on the treatment results, expert-led initiatives have been put into place, in cooperation with the board certification system, in order to improve the treatment results. Instead of exposing the poor performing facility or the surgeons, facility improvement should be supported anonymously, and a Plan-Do-Check-Act cycle needs to be facilitated. This initiative is beneficial for promoting the quality improvement of future medical treatments. The effectiveness of public reporting, in which the facility names are made public, and of pay for performance, wherein the medical fees are adjusted according to the facility’s performance, have received mixed reviews. In Japan, the initiative to improve future medical treatments has been planned. The initiative will begin with aims to optimize the surgical indications for LS and to establish a safe education system for this procedure that is in cooperation with expert-led quality improvement.

### Limitations

Recent trends in the therapeutic indications have a varying impact on the treatment results, depending on associated factors, such as whether the technique should be introduced in its infancy and whether the indication is established. The findings of the present study are limited in their generalizability, as the situation may change over time, even within the same geographical region. An ongoing evaluation on the effect of the introduction and dissemination of a medical technique over time remains important [22]. In subsequent studies, we will report on the results of an analysis of treatment indications; evaluate the effects of using a laparoscopic approach; and discuss the learning curve related to the annual number of cases performed, including its association with the complication rate. A care must be taken when interpreting the learning curve in relation to the annual number of cases in the present study. In Japan, facility surgeons share case experiences in a conference, and multiple surgeons attend each operation. Therefore, as shown in a previous study, the treatment results can become stable despite a relatively small number of cases [23,24]. Also, in the current study, we only analyzed surgical procedures of four aforementioned procedures. The other procedures, such as cholecystectomy, will need additional assessment in the future. Additionally, in regions where the operation and conference systems differ, cut-off points are likely to be different as well. As another limitation, the end point was limited to short term overall survival. Additionally, our analysis our analysis did not include surgeon-level factors such as their past procedural experience, nor did we include the presence of Japanese endoscopic surgical skill certification in both hospital and surgeon levels which may have acted as confounding factors. We also acknowledge the potential presence of residual confounding; while we adjusted for baseline clinical factors previously identified as predictors of short term mortality after these procedures, factors such as patient’s social economic status or hospital-level factors such as its rurality or academic status were not included in the study. Finally, O/E value based analysis might be sensitive to the skewness in the distribution, especially since there is no standard for what value is too high for the O/E value. The consistency in the results of the analyses using O/E as both continuous and categorical variables may support the robustness of our findings.

## Supporting information

S1 Fig Distribution of hospital annual volume for each surgery. (DOCX)

S2 Fig Hospital rate of LS for each procedure (restricted to hospital annual volume 10 and over). (DOCX)

S3 Fig Distribution of standardized LS ratio. (DOCX)
