# Supplementary material for: Association between institutional procedural preference and in-hospital outcomes in laparoscopic surgeries; Insights from a retrospective cohort analysis of a nationwide surgical database in Japan
Source: PLoS One. 2018 Mar 5;13(3):e0193186. doi: 10.1371/journal.pone.0193186 (PMC5837082; doi:10.1371/journal.pone.0193186)
Supplement: S2 Fig — (DOCX) [file pone.0193186.s002.docx]

**S2 Fig. Hospital rate of LS for each procedure (restricted to hospital annual volume 10 and over).**
